# Chitosan with Natural Additives as a Potential Food Packaging

**DOI:** 10.3390/ma16041579

**Published:** 2023-02-14

**Authors:** Karolina Stefanowska, Magdalena Woźniak, Renata Dobrucka, Izabela Ratajczak

**Affiliations:** 1 Department of Chemistry, Faculty of Forestry and Wood Technology, Poznan University of Life Sciences, Wojska Polskiego 75, 60625 Poznań, Poland; 2 Department of Industrial Products and Packaging Quality, Institute of Quality Science, Poznań University of Economics and Business, al. Niepodległości 10, 61875 Poznań, Poland

**Keywords:** chitosan, food packaging, antimicrobial activity, mechanical properties, composite film

## Abstract

Recently, the development of materials based on natural polymers have been observed. This is the result of increasing environmental degradation, as well as increased awareness and consumer expectations. Many industries, especially the packaging industry, face challenges resulting from legal regulations. Chitin is the most common biopolymer right after cellulose and is used to produce chitosan. Due to the properties of chitosan, such as non-toxicity, biocompatibility, as well as antimicrobial properties, chitosan-based materials are used in many industries. Many studies have been conducted to determine the suitability of chitosan materials as food packaging, and their advantages and limitations have been identified. Thanks to the possibility of modifying the chitosan matrix by using natural additives, it is possible to strengthen the antioxidant and antimicrobial activity of chitosan films, which means that, in the near future, chitosan-based materials will be a more environmentally friendly alternative to the plastic packaging used so far. The article presents literature data on the most commonly used natural additives, such as essential oils, plant extracts, or polysaccharides, and their effects on antimicrobial, antioxidant, mechanical, barrier, and optical properties. The application of chitosan as a natural biopolymer in food packaging extends the shelf-life of various food products while simultaneously reducing the use of synthetic plastics, which in turn will have a positive impact on the natural environment. However, further research on chitosan and its combinations with various materials is still needed to extent the application of chitosan in food packaging and bring its application to industrial levels.

## 1. Introduction

Nowadays, more and more often, we struggle with the negative effects of improper disposal of products made of plastics at the end of their life cycle [1,2,3]. According to research, about 359 million tonnes of plastic was produced in 2018, and 14.5 million tonnes were released into the oceans [3], its mass as much as six times that of plankton, which is the basis of the food chain of many marine animals [1]. Plastics can accumulate chemicals such as polychlorinated biphenyls (PCBs) and dichlorodiphenyltrichloroethane (DDT), which bioaccumulate and are absorbed with food down the food chain [1,2]. Moreover, plastic waste accumulated in the waters limits access to light and oxygen, which contributes to the degradation of coral reefs [3,4,5,6].

Plastic food packaging can also have a negative impact on human health [7]. Substances such as bisphenol A (BPA) and phthalates pose a threat to health. In 2011, the European Union banned the use of BPA in children’s plastic bottles [7]. Research shows that BPA is transferred from packaging to packaged food, primarily due to mechanical damage to the packaging, in contact with acidic and sweet foods and foods with a high fat content [7]. BPA affects the work of the human endocrine system; its presence is associated with a negative influence, among others, on women’s health, such as through the occurrence of polycystic ovary syndrome, which is diagnosed with increasing frequency, and with problems with getting pregnant and maintaining pregnancy [8,9]. In addition, phthalates can easily get from packaging to packaged food; their high content is mainly observed in highly processed foods. They have a negative effect on reproduction in both women and men, contribute to the occurrence of insulin resistance and type II diabetes, and are also associated with the occurrence of skeletal anomalies, asthma, and cancer [10,11,12].

In the coming years, the packaging industry will have to make many changes resulting from the regulations set out in Directive 2019/904 of the European Parliament and of the Council [13] on the reduction of the environmental impact of certain plastic products. This directive is related to the transition of the European Union to a circular economy and is the result of the observed environmental degradation, as well as increased awareness and consumer expectations [14]. To implement the assumptions resulting from the directive, the circular economy, and the zero-waste approach, packaging producers are facing enormous changes [15,16]. The answer to these challenges is the search for new natural materials which can be applied in the packaging industry.

**Figure 1:**
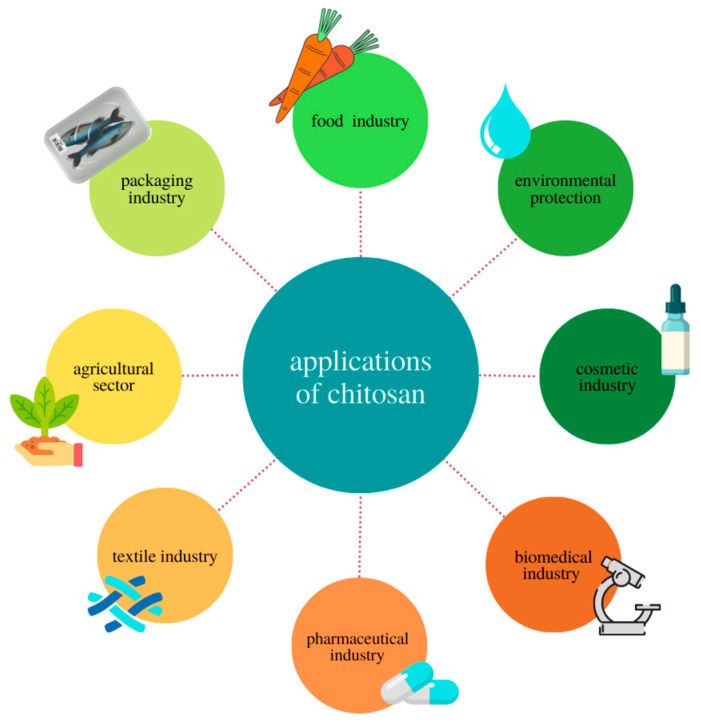
Application of chitosan.

Chitosan is a natural polysaccharide with multiple beneficial properties, such as non-toxicity, biocompatibility, biodegradability, as well as antimicrobial properties. In addition, chitosan is obtained from shellfish shells, which are a by-product of the food industry [17,18,19]. Therefore, it is used in many industries, such as medicine, agriculture, textiles, the food industry, and environmental protection, as shown in Figure 1.

In medicine, chitosan-based materials are used as a dressing material, causing faster regeneration of injuries and burn wounds [20,21]. In the field of skin and bone tissue engineering, chitosan is used to produce a wide range of materials, from membranes and hydrogels to sponges and fibers [22,23]. Moreover, literature reports have shown that chitosan and chitosan-based materials can be used as various types of drug carriers, including anti-cancer drugs, nasal drugs, gene delivery drugs, prenatal drugs, or ocular drug systems [24]. In cosmetology, scientists are investigating the possibility of using chitosan in the production of anti-aging creams or for protection against UV radiation [25,26]. Chitosan-based materials are also widely used in the field of agriculture. These materials can be used to protect plants against the harmful effects of microorganisms, stimulate plant growth, or control the release of agrochemicals [23,27,28]. Chitosan materials are also used in environmental protection, including cleaning wastewater from heavy metals such as Ni, Cu, As, Cd, and Pb [29,30]. Researchers are also increasingly interested in the possibility of using chitosan materials in the food and packaging industries, where new solutions are sought that will be environmentally friendly and allow for the replacement of the plastic packaging produced so far [17]. Research has shown that it is possible to produce chitosan films by various methods, such as casting or extrusion. Chitosan films can be additionally modified in order to increase their antioxidant, antimicrobial, and mechanical properties or to improve the barrier and optical properties [17,19,31]. Literature reports described the applications of chitosan films for packing various food products, e.g., meat, fruit, and vegetables, and the research showed that chitosan films successfully extend the shelf life of products [17,32,33].

In order to obtain a comprehensive description of biodegradable chitosan-based materials and the possibilities of their application in the packaging industry, it is necessary to look at their antimicrobial, antioxidant, mechanical, barrier, and optical properties. This review describes the current literature on chitosan-based materials and their potential use in the food packaging industry. The article presents literature data on the most commonly used natural additives, such as essential oils, plant extracts, or polysaccharides, which can improve the above-mentioned properties.

## 2. Chitosan as a Future of Plastic

Chitosan is obtained by the alkaline N-deacetylation of chitin, which is the second most common biopolymer after cellulose [34,35,36]. Chitin can be found in the exoskeleton of crustaceans, insects, and in the cell walls of fungi [37]. It is composed of β-glucosamine molecules linked by β-1,4-glycosidic bonds. Chitin has a linear structure of the chain, and it is characterized by numerous intra- and intermolecular interactions [38,39].

**Figure 2:**
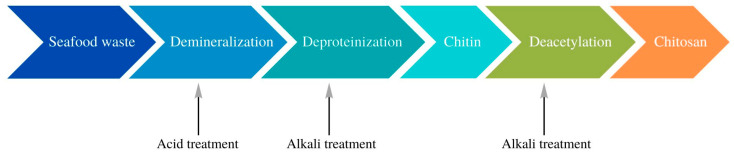
The scheme for obtaining chitin and chitosan in the chemical extraction process.

Chitin exhibits greater mechanical strength compared to cellulose, which is the result of stronger hydrogen interactions [38,39]. Moreover, compared to cellulose, it is characterized by higher crystallinity and stronger hydrogen bonds, which results in its lower solubility and is associated with the presence of acetylamine functional groups in the chitin structure [39,40]. Chitin, due to its high biocompatibility and non-toxicity, is of great interest in the field of medical science. It is widely researched in terms of its use as hydrogel dressings or modern carriers in drug delivery systems [41,42]. Literature reports show that chitin, thanks to its high biocompatibility, can be an important material in the field of tissue engineering [43,44]. The antibacterial and antifungal properties of chitin are used in the production of dressing materials [45]. Moreover, literature data indicated that chitin, in its native form, can also be applied in the field of electrochemistry, where it is usually used as a substitute for synthetic polymers and acts as a matrix for hydrogel electrolytes [46,47,48]. Difficulty processing chitin and its solubility constitute a factor limiting the use of chitin on a larger scale. Therefore, chitin is primarily used to obtain chitosan, a more reactive derivative that is easier to process [48]. Chitin, on an industrial scale, is most often obtained by chemical extraction. This type of extraction involves the use of a strongly alkaline solution, such as sodium hydroxide. This process results in the disintegration of polymer chains and the formation of chitosan with a high degree of deacetylation [49]. The chitin-containing material is subjected to washing, drying, and powdering. The extraction process includes three main stages–deproteinization with an alkaline solution, demineralization with an acidic solution, and decolorization [50]. Deproteinization involves breaking the chemical bonds between proteins and chitin. This process uses strong acids and bases and high temperatures, which consume large amounts of energy and produces wastewater with a high chemical concentration, which must be subjected to appropriate treatment and neutralization. The next step is demineralization. In this process, minerals such as calcium carbonate are removed. The demineralization process is also carried out by the use of concentrated coffees, most often sulfuric acid, hydrochloric acid, acetic acid, or nitric acid. Discoloration is an additional step that leads to the elimination of color if the process is intended to produce a colorless product. For this purpose, solvents such as acetone, sodium hypochlorite, or hydrogen peroxide are used [49]. The scheme for obtaining chitin and chitosan is shown in Figure 2.

**Figure 3:**
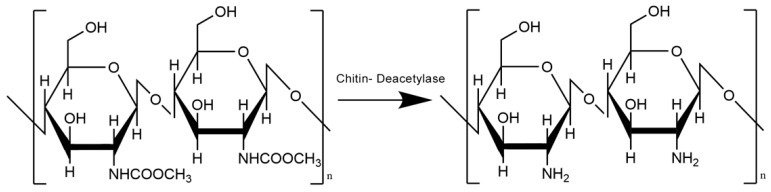
Chitin deacetylation and chemical structure of chitosan.

Chitosan is obtained from chitin by the alkaline N-deacetylation and leads to chitosan with various deacetylation degrees [34,35]. The degree of deacetylation (DA) is the ratio of the number of N-acetyl-D-glucosamine units present in the polymer chain to the total content of N-acetyl-D-glucosamine and β-(1,4)-D-glucosamine units in it. A value equal to 0.50 is taken as the DA cutoff value between chitin and chitosan [34,35]. The chemical structure of chitosan formed in the deacetylation process from chitin is shown in Figure 3.

On an industrial scale, chitosan is most often obtained by heterogeneous, basic chitin deacetylation. The most commonly used precursor is α-chitin obtained from the shells of sea arthropods, which are waste from the food industry [18]. The type of chitosan obtained depends on the parameters of the preparation process, the concentration of the solvents used, temperature, and duration of the process [18,51,52,53].

Chitosan is an odorless solid. It comes in the form of flakes or powder, and its color ranges from white to pale yellow or gray. The chemical composition of the polymer chitosan chain is very similar to the chitin chain, but the increase in the proportion of β-(1,4) -D-glucosamine units causes the reconstruction of the hydrogen bond network, both inside and intermolecular. The presence of a greater number of more reactive amino groups and changes in the interactions between the chains make the physicochemical properties of chitosan different from the physicochemical properties of chitin [54,55]. Due to the degree of acetylation reduction, chitosan is characterized by better solubility than chitin. Chitosan solubility is also influenced by factors such as the source of production, molecular weight, fragmentation, or crystalline form [56,57]. Chitosan, like chitin, is insoluble in water and organic solvents; therefore, acidic solutions with a pH below 6.3 are used to produce film-forming chitosan solutions [36,58,59,60]. Chitosan flakes intake water form a suspension and swell. The reason is the still strong interactions between chitosan molecules and water molecules between polymer layers, which prevents the hydration of polymer chains [55]. In the deacetylation process, primary amino groups are formed in the chitosan structure, which makes chitosan a strong base, and under the influence of low-concentrated acids, the chitosan molecule is easily ionized, and a cationic polyelectrolyte is formed [60]. In this form, the ionized amino groups of chitosan interact with the anions of the solvent, which results in the loosening of the ordered chitosan structures, and then the breaking of the hydrogen bonds and transferring the polymer to the solution [58,60].

**Figure 4:**
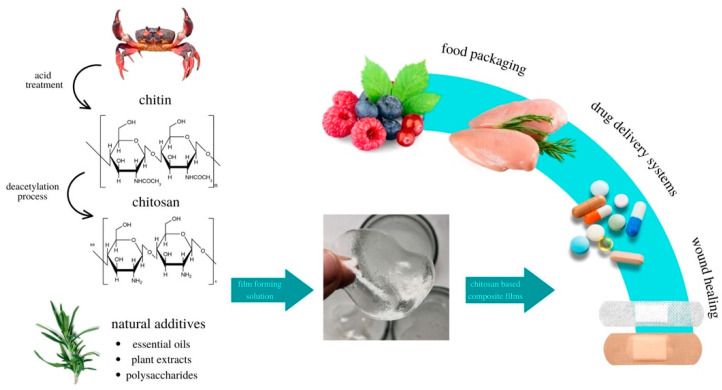
Usage of chitosan for film production and its potential applications.

Due to properties such as non-toxicity, biocompatibility, and microbiological activity, but also properties that result from the modification of the polymer chain, chitosan is the subject of many studies in terms of potential applications [31,61]. Literature data indicate that chitosan is used in many industries such as agriculture, food industry, environmental protection, and medicine [31,54,62,63,64,65,66,67]. Figure 4 shows the possibility of using chitosan to produce chitosan films and its potential applications.

**Figure 5:**
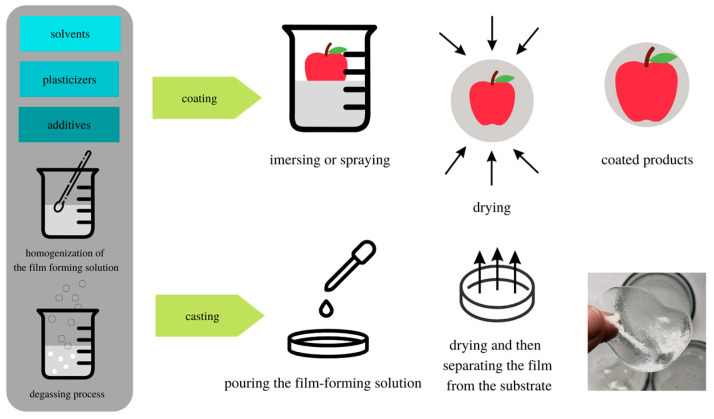
Scheme presenting obtaining process of chitosan-based films and coatings.

Literature data indicate a potential application of chitosan in the packaging industry [68]. It has many advantages in the context of the production of traditional food packaging films–it is non-toxic, biodegradable, bio-functional, biocompatible, and it can be modified [68]. Moreover, chitosan exhibits antibacterial and antifungal properties [18,31,69,70,71]. The chitosan samples available on the market include samples of various molecular weights and degrees of deacetylation, which affects the properties of the obtained films [14,72]. The good film-forming properties of chitosan allow the production of films with good mechanical and microbiological properties by coating or casting technique [16,72]. The scheme for obtaining chitosan-based films and coatings is presented in Figure 5.

## 3. Natural Additives for Chitosan Matrix

Chitosan has many valuable features that make it a serious candidate for use as a matrix in natural and biodegradable composite materials with various applications. In addition, its possibility of modifying or adding many different compounds and substances with significant biological, mechanical, and barrier properties affects the possibility of obtaining materials with increased parameters, thus increasing the possibility of using the obtained chitosan-based products. Studies have shown that despite the fact that chitosan solutions show antimicrobial properties against a number of strains of pathogenic bacteria, unfortunately, chitosan films obtained from these film-forming solutions do not have such properties. This may be due to the binding of chitosan in the films and the impossibility of direct interaction of its chains with microbial cells [73]. Therefore, appropriate and natural additives such as essential oils or plant extracts are sought, which could ensure the appropriate microbiological activity of chitosan films and act synergistically in order to obtain a film with appropriate parameters. In order to improve the properties of these parameters, chitosan films are also modified using synthetic additives, such as metal nanoparticles [74] or built-in sensor elements [75].

Due to the risk to health resulting from the use of synthetic additives such as metal nanoparticles in food packaging or dressings materials, more and more attention is focused on natural additives that are non-toxic, biodegradable, and safe for the environment [74,75,76,77]. Among the additives of natural origin mixed with chitosan in order to obtain a film intended for packaging and storing food, mention should be made, among others, to essential oils, plant extracts, or polysaccharides. This section described the physicochemical, biological, and mechanical properties of chitosan films with natural additives.

### 3.1. Essential Oils

Essential oils are a complex mixture of volatile components that are synthesized by living organisms and can be obtained through water, steam, and dry distillation [77]. Research to date has shown that essential oils are used by the organisms that produce them for defense, signaling, or as part of their secondary metabolism. Essential oils are an important raw material for renewable natural products [77,78]. Thanks to valuable properties such as bactericidal, virucidal, and fungicidal activity, essential oils are a valuable raw material with various applications [79]. Essential oils are commonly used in the food, perfume, cosmetic, and pharmaceutical industries [77,79], and according to the research data, they can also be used as an additive to chitosan films [80,81,82]. Essential oils are found primarily in aromatic plants. The families with numerous species rich in essential oils include, among others, *Apiaceae*, *Asteraceae*, *Cupressaceae*, *Hypericaceae*, *Lamiaceae*, *Lauraceae*, *Myrtaceae*, and *Pinaceae* [77]. They include two groups of ingredients of different origins. The main groups are terpenes and terpenoids, and the second group includes aromatic and aliphatic substances [80,81].

A valuable property of essential oils because they are used as a natural additive to chitosan films is mostly their antibacterial effect. The structure of the cell wall of gram-positive bacteria allows easy penetration of hydrophobic molecules into cells; therefore, the molecules of essential oils can affect both the cell wall and the cytoplasm. In the case of gram-negative bacteria, the effect of essential oils is more limited due to the more complex structure of the cell wall [82,83]. The effect of essential oils on bacteria depends on their chemical composition and concentration: at low concentrations, they can interfere with the enzymes responsible for energy production, and at higher concentrations, they can denature proteins [83,84]. Studies have shown that the antibacterial activity of essential oils depends on the number of individual compounds present in a given oil. High concentrations of cinnamaldehyde or eugenol provide good antibacterial activity [83,84]. Monoterpenes and phenols that occur in essential oils obtained from, among others, sage, thyme, or rosemary have antibacterial, antifungal, and antiviral activity [85]. The good antimicrobial activity of some essential oils is used to improve the antimicrobial properties of chitosan films [86,87]. Unfortunately, literature data show that adding some essential oils, such as oregano oil, to the chitosan film matrix can adversely affect its mechanical properties [86]. The most frequently used essential oils as an additive to chitosan films and their influence on film properties are presented in Table 1.

### 3.2. Plant Extracts

Plant extracts are obtained from various parts of plants–roots, leaves, bark, fruit, and seeds [74]. Plant extracts can be used as additives to chitosan films and other materials produced from biomass and applied in the packaging industry to improve their properties [76]. As an additive to chitosan films, they have a wide spectrum of activity, from improving antioxidant and antimicrobial properties to the impact on the anti-corrosive properties of films and their ability to protect against UV radiation [74,86,87,88,89]. Despite many advantages when used as an additive to the matrix of chitosan films, such as low price, environmental friendliness, and widespread availability, plant extracts also have disadvantages [77]. One of them is the instability of the plant extracts. They are biodegradable, which enables their long shelf life [96,97]. Some components of plant extracts are oxidized or evaporated. It is also important to choose the right solvent for the extraction in order to obtain as many active ingredients as possible from a given plant [98].

The plant extracts contain phenolic compounds, which are responsible for their antibacterial properties. These properties are related to the presence of a hydroxyl group in phenolic compounds [99,100] and can be used to improve the antibacterial properties of chitosan films [101,102,103,104]. The hydroxyl group disrupts the activity of the microbial cell membrane–it causes its disruption and leakage of cell components [92]. The most frequently used plant extracts as additives to chitosan films and their influence on their properties are presented in Table 2.

### 3.3. Polysaccharides

The polysaccharides most often combined with chitosan to obtain films with better mechanical and antimicrobial properties compared to pure chitosan films includes nanocellulose, starch, pectin derivatives, and agar [32,109,110,111].

Nanocellulose is a biodegradable nanofiber with low weight and low density. It is characterized by exceptional strength properties–high tensile strength. Nanocellulose is an effective material for the strengthening of polymers due to the interactions between nano-sized materials because they form a network connected by hydrogen bonds. An important factor is the good dispersion of nanofibers in the matrix [112,113]. There are three types of nanocellulose: crystal nanocellulose (CNC), cellulose nanofibers (CNF), and bacterial nanocellulose (BNC). These types of nanocellulose are analogous in composition but differ in morphology, particle size, or crystallinity due to different sources and extraction methods [114]. Two types of nanocellulose, CNC and CNF, are the most often used as an additive to chitosan film matrices. Research has shown that the addition of CNC has a positive effect on the microbiological properties of chitosan films [115]. The chitosan film showed bactericidal activity against *S. aureus* and *E. coli*; in terms of antifungal activity, the chitosan films showed fungicidal activity against *C. albicans*. The bactericidal and fungicidal effects were maintained after the incorporation of CNC into chitosan films. Moreover, with the incorporation of 10% and 25% CNC, the antimicrobial activity was total [115]. The influence of the addition of nanocellulose on the mechanical strength of chitosan films was also investigated. After incorporating the CNC into chitosan films, a gradual increase in tensile strength was observed from 7.98 to 8.93, 13.0, and 25.3 MPa, corresponding to the concentration of the CNC. However, this difference was only statistically significant when 50% by weight of the CNC was added [115]. Mechanical properties and water vapor barrier properties of chitosan films were also affected by the addition of CNF. A composite film with 15% CNF was comparable to synthetic polymers in terms of strength and stiffness, but their elongation and water vapor barrier were weaker [116]. Research shows that different types of nanocellulose have different effects on the mechanical strength of chitosan films. The different elongation at break was observed for CNC-chitosan (2%) and CNF-chitosan (4%) blends. The higher elongation at break and tensile strength was observed for the CNF-chitosan blends in comparison to CNC-chitosan samples [117].

Starch, due to its cheap price, edibility, biodegradability, and easy access is a good raw material for the production of biodegradable films. Starch is a plant polysaccharide composed of glucose units linked by α-glycosidic bonds; in plants, it is an energy store [118,119,120]. Amylose is responsible for the film-forming properties of starch solutions [119,121,122,123]. To obtain starch film-forming solutions, the starch must be heated in excess of water. During heating, the crystal structure is broken, and water molecules interact with the hydroxyl groups present in amylose and amylopectin. In the temperature range of 65–90 degrees Celsius, the starch gelatinization process takes place, which is an irreversible process [119,124]. Unfortunately, fully starch-made foils have poor mechanical properties and high-water vapor permeability. These properties make the applicability of starch films limited [125,126]. The mixing of starch with other polymers, such as chitosan, had a positive effect [127,128]. The advantage of starch films is that they show very good oxygen barrier properties, which is related to their highly ordered network structure of hydrogen bonds [36]. Literature reports indicated that both starch films with and without the addition of plasticizers exhibit good oxygen barrier properties [129]. In order to exploit the advantages of starch films, such as very good oxygen barrier properties, and reduce their disadvantages, research has been carried out on the possibility of using starch-chitosan blends to obtain good quality films [110,111]. Literature data show that numerous studies have been carried out to produce chitosan-starch composite films [129,130,131,132,133]. To some extent, starch-chitosan interactions will inhibit excessive interactions of chitosan with additives such as, e.g., murta leaf extract, which allows for obtaining films with better parameters by limiting excessive chitosan cross-linking by the additive [132].

Pectins are another polysaccharide used to produce composites with chitosan [134,135,136,137,138,139,140,141,142]. Pectins are the main component of plant cell walls, and they ensure the integrity and stiffness of tissues [139]. They are one of the most complex macroparticles found in nature. For commercial production of pectins, mainly apple and citrus peels are used [36,139,140]. The properties of pectin texture and stability depend on their molecular weight, degree of esterification, and the method of extraction [36,141]. Pectin-based films have good gas permeability properties but are not resistant to water [136]. It is known that pectins themselves have antimicrobial properties [36,134]. Therefore, the properties of films obtained with the use of pectins and chitosan were investigated. Research showed that the presence of pectins in the chitosan-pectin composite film increases the antimicrobial activity against *E.coli*, *S. aureus,* and *Helicobacter pylori* compared to the pure chitosan film [139]. In addition, composite films made of chitosan and pectin have a higher tensile strength value and a lower value for elongation at break compared to pure chitosan films [141].

## 4. Selected Properties of Chitosan-Based Materials

Packaging materials made of natural polymers, thanks to the possibility of using appropriate functional additives, often have better properties than traditional plastic packaging. Packaging materials are designed to inhibit the growth of microorganisms or limit unwanted chemical reactions, which contributes to extending the shelf life of the packed food [143,144,145,146]. When producing packaging films from natural polymers such as chitosan, many factors should be taken into account, such as, for example, the selection of an appropriate solvent or the molecular weight of the chitosan used [147,148]. They have an impact on the properties of the obtained films and their suitability in the context of their potential application for food packaging [148].

Research shows that the selection of appropriate chitosan can have an impact on the properties of chitosan films. Numerous studies have been carried out using chitosan with different molecular weights and degrees of deacetylation for film production [148,149]. The results of the research show that films obtained with the use of high molecular weight chitosan are characterized by better mechanical, barrier, and antimicrobial properties compared to films for which chitosan with a lower molecular weight was used. In turn, chitosan with low molecular weight is characterized by greater antioxidant activity than with high molecular weight [148,149,150,151].

The properties of chitosan films can also be improved by using appropriate natural additives. For this purpose, additives with antimicrobial and antioxidant properties are introduced into the film-forming solutions [152,153,154,155]. This section describes the properties of chitosan films that are relevant to their potential use as food packaging materials and describes the possibility of using natural additives to improve these properties. Characteristics of chosen natural additives and their division into categories are presented in Section 3.

### 4.1. Antioxidant Properties

Antioxidant studies of materials with potential use for food packaging are particularly important in the context of the possibility of limiting the oxidation of food that occurs during its storage. Food oxidation is a complex process. Lipid molecules and oxygen take part in the oxidation process. The oxidation reaction leads to the formation of free radicals. During the food storage process, oxidation of the lipid particles occurs, resulting in an unfavorable change in the taste and appearance of the packaged food. In addition to the negative impact on the quality of packaged products, the oxidation process also causes the loss of biological activity of lipophilic bioactive particles that naturally occur in food or are specially included in it to increase its health value [156]. The most common natural antioxidant compounds include vitamins E, C, and A, bioflavonoids, carotenes, and hydroxycinnamates [157]. Natural antioxidants have different mechanisms of action; they bind metal ions, scavenge free radicals, decompose peroxides, or act synergistically [158]. Phenolic compounds show two mechanisms of action by inactivating free lipid radicals and preventing the decomposition of hydroperoxide into free radicals. Phosphoric and citric acids bind heavy metals into inactive compounds, causing metal chelation. Carotenes are responsible for the transformation of singlet oxygen into a triplet. Proteins and amino acids reduce hydroperoxide in a non-radical way. Citric acid is an example of a compound with a synergistic effect, which promotes the action of other antioxidants [159].

Literature data show that chitosan films can be used as active packaging to prevent food oxidation. Natural additives rich in phenolic compounds are often included in the chitosan matrix in order to intensify the antioxidant properties of obtained films [160]. Antioxidants are incorporated into packaging materials to inhibit the food oxidation reaction. Antioxidants neutralize singlet oxygen, reduce hydrogen peroxide, and quench free radicals, inhibiting the food oxidation process [145,161,162]. Rich sources of natural antioxidants that can be combined with chitosan film-forming solutions are plants; therefore, plant extracts are widely used as additives in active packaging materials [163,164].

One plant extract used as an additive to increase the antioxidant properties of chitosan films is rosemary extract [165,166]. Rosemary (*Rosmarinus officinalis*) is widely used in food for its taste and medicinal properties. The rosemary extract contains flavones such as apigenin, genquanin, phenolic diterpenes, e.g., carnosic acid, rosmanol, carnosol, and phenolic acids–caffeic acid, and rosmarinic acid which are responsible for increasing the antioxidant activity of films [166,167]. Oregano extract was also used as an additive to polysaccharide packaging materials to increase their antioxidant properties [168,169,170]. Oregano (*Origanum vulgare*), like rosemary, is a plant valued for its organoleptic and health properties. The extract obtained from oregano has both antimicrobial and antioxidant properties. The main substances responsible for these properties include carvacrol, thymol, *p*-cymene, and terpenes [86,168,171,172]. Green tea is also a rich source of antioxidants; therefore, it is commonly used as an additive to chitosan films [94]. It is rich in phenolic compounds such as catechin, theaflavin, and thearubigin. These compounds scavenge reactive oxygen and nitrogen species [172,173]. Green tea extract is used as an additive to lipid-containing foods because it delays their oxidation and extends the shelf life of the food [174]. Research has shown that epigallocatechin and epigallocatechin gallate has the highest antioxidant activity in green tea extracts [175]. Literature data shows that also ginkgo biloba leaf extract has good antioxidant properties and can be used as an additive for chitosan film matrix to improve its antioxidant properties [176]. This extract contains 22–27% flavonoid glycosides and 5–7% terpene lactones which are responsible for the antioxidant properties [176].

Apart from extracts, literature data show that other compounds, for example, anthocyanins, can also be used as additives with antioxidant properties to the chitosan matrix [86,95,162,166,176]. Research shows that anthocyanins act as an oxygen compressor and can be used as an antioxidant additive to packaging materials [177]. Anthocyanins such as curcumin contain many phenolic hydroxyls, which can effectively deliver hydrogen donors to free radicals, thus blocking a chain reaction and improving antioxidant activity [178]. Studies have shown that chitosan films with the addition of curcumin showed higher antioxidant activity compared to pure chitosan films and reduced the oxidative degeneration of packaged foods, especially foods rich in oils [179].

### 4.2. Antimicrobial Properties

Numerous studies show that despite the good antimicrobial activity of chitosan, chitosan films do not show or show very poor inhibitory effects against microorganisms [155,156,157,158,159,160,161]. Such an effect of chitosan films may be related to the fact that chitosan does not diffuse from the films, as a result of which the growth of only those microorganisms that are in direct contact with active sites of chitosan is inhibited. In addition, the antimicrobial effect of chitosan requires positively charged amino groups of chitosan, which can react with the anionic groups on the surface of microbial cells, inhibiting mRNA and protein synthesis and creating an external barrier that prevents access to the necessary nutrients for microbial growth [161]. Due to poor antimicrobial properties, research is ongoing to include additives in the chitosan matrix in order to improve these properties.

Substances with antimicrobial activity are desirable additives to chitosan films. By inhibiting the growth of pathogenic and rotting microorganisms, it is possible to extend the shelf life of packaged food products. Plant extracts, among others, can be used as natural antimicrobial additives from barberry, saffron, garlic, red cabbage, mango leaf, pomegranate rind, or essential oils such as those obtained from cloves, oregano, thyme, or rosemary [101,162,163,164,165,166]. Antimicrobials can be incorporated inside the biopolymer matrix or as a coating on their surface [163]. When using natural additives, the appropriate film production technique must be adapted. Some of these substances are sensitive to higher temperatures, and their effect may be limited in the production process [167].

#### 4.2.1. Antibacterial Properties

One of the main causes of food spoiling too quickly is microbial contamination during processing or transport [180]. Therefore, new and more ecological solutions are being sought to enable food packaging and simultaneous protection against the harmful effects of microorganisms. Plant extracts or oils that can be used as additives are rich in various classes of antibacterial compounds [158]. Depending on the content of individual components, their operation may be based on different mechanisms. Inhibition of the growth of bacterial colonies may be caused by damage to the structural elements of bacterial cell membranes. Damage to the cell membrane reduces or completely prevents the transport of nutrients so that bacterial cells cannot develop. Flavonoids affect the integrity of the cell membrane of the cytoplasm, which causes the release of ions and macromolecules outside the bacterial cell, which results in its damage and death. In the case of essential oils, substances belonging to the group of alcohols are often distinguished in their composition, which causes the denaturation of bacterial proteins [181].

The research shows that there are many natural additives to the chitosan matrix that can increase the antibacterial activity of the obtained films. For example, literature data have shown that the addition of rosemary extract into the chitosan film matrix increases the antibacterial activity of the film against *E. coli* and *S. aureus,* which are common pathogenic bacteria [95]. Additionally, the incorporation of turmeric extract increased the antibacterial activity of chitosan films against *S. aureus* [127]. Bioactive compounds that are responsible for the biological activity of turmeric extract are primarily sesquiterpenes: bisabolans, guaians, germacranes, carans, elemans, spironolactones, selinates, santalans, and caryophyllates [167]. Another natural substance incorporated into the chitosan matrix that increased the antibacterial activity of films is thyme oil. The main bioactive compounds that are responsible for the biological activity of thyme oil are thymol, *p*-cymene, γ-terpinene, and linalool [168]. Chitosan films with the addition of thyme oil showed higher activity against *Klebsiella pneumoniae, E. coli*, *P. aeruginosa,* and *S. aureus* in comparison with pure chitosan films [97,98,169,170].

#### 4.2.2. Antifungal Properties

Studies show that the antifungal activity of chitosan films can also be enhanced by applying additives to the matrix of chitosan films. The indicator strains most frequently used in antifungal activity tests are *A. niger* and *C. albicans.*

Literature reports have shown that the use of the addition of cinnamon and ginger oils increases the antifungal activity of chitosan films against *A. niger* [182]. The higher concentration of these oils in the film matrix, the greater the antifungal activity of the films [182,183]. The main component of cinnamon oil, which is responsible for its antifungal properties, is cinnamic aldehyde [184]. In the case of ginger oil, the main antifungal component is α-zingiberen [185]. However, in both, essential oils contain a wide variety of volatile compounds, including terpenoids, esters, aldehydes, ketones, acids, and alcohols that act synergistically [186].

### 4.3. Mechanical Properties of Chitosan Films

Chitosan films in many areas, such as biological activity, antioxidant properties, or biodegradability, are more favorable than traditional plastic packaging, but research is still ongoing to improve their mechanical properties [68,187,188]. The mechanical properties of materials used in the packaging industry are extremely important to fulfill their utility function [189].

The tensile strength of the films obtained on the basis of chitosan is influenced by its molecular weight and the degree of deacetylation [72,190,191,192,193]. Films produced from chitosan with a high deacetylation degree showed higher tensile strength and elongation than films with a low deacetylation [194]. The results of Hsu et al. [195] indicated that the production of chitosan films from biopolymer with a similar degree of deacetylation but higher molecular weight led to a higher tensile strength of obtained films. Literature data indicate that the mechanical properties of the obtained chitosan films are also influenced by the type of solvent used in the production process. Chitosan films made with acetic acid show higher breaking strength than films with lactic, malic, or citric acid as a solvent. The mechanical properties are also influenced by the drying and storage temperature of the obtained films, as well as the type of plasticizer [193,196,197,198,199,200]. Research has shown that the tensile strength of chitosan films for acetic acid production was used as a solvent increase during storage at room temperature [193,196]. The use of choline chloride-based deep eutectic solvent with malonic acid as a plasticizer of chitosan resulted in a significant increase in the elasticity of chitosan film [201]. Additionally, the addition of turmeric to chitosan film significantly increases the tensile strength of the film [102]. In turn, cinnamon essential oil decrease elongation at the break of chitosan films [91]. The addition of ethanolic extracts of propolis (EEP) also affects the tensile strength and elongation at the break of chitosan films [202]. The results of Siripatrawan et al. [202] showed that the elongation at break of the chitosan films increased when propolis concentrations increased from 0 to 10% but significantly decreased when 20% propolis was added. In turn, the tensile strength of propolis-chitosan films increased when the concentration of propolis extract increased from 0–20% [202]. Studies have shown that the incorporation of boswellic acid into the matrix of chitosan films significantly enhanced the tensile strength, Young’s modulus, and elongation at break compared to films without this addition [203]. The use of glycerol improves mechanical properties, including tensile strength and elongation at break [19,188,193]. The application of olive oil, corn oil, and oleic acid as a plasticizer in chitosan-based composites affected their mechanical properties [204,205,206]. According to the research, cellulose nanofibers (CNF) and lignin nanoparticles can improve the mechanical and water vapor barrier properties of chitosan films [116,117,207,208,209].

### 4.4. Barrier Properties of Chitosan Films

The barrier properties of biopolymer food packaging materials are important to maintain the shelf life and nutritional value of packaged products [210]. The barrier properties of chitosan materials play an important role in the storage of products and translate into their ability to preserve food [211]. Reducing the penetration of water vapor and oxygen through the packaging material is necessary to limit the development of microorganisms and the decomposition of some active substances [212]. The chitosan itself, which was used for their production, has a very large impact on the barrier properties–its molecular weight, degree of deacetylation, but also modifications of chitosan films, additives, or plasticizers used [213]. As the molecular weight of chitosan increases, the water vapor transmission coefficient (WVTR) also increases [193]. An important aspect in the context of assessing the barrier properties of chitosan films is the method of their production. Studies have shown that the way films are dried is important. Thermally dried films showed a lower WVTR value compared to air-dried films [214]; as the storage temperature decreases, the water vapor consumption and permeability of chitosan films increase [193]. The addition of propolis extract (EEP) reduces the permeability of water vapor and oxygen. The higher concentration of propolis in the chitosan films, the lower permeability [202]. The addition of clay to the matrix of chitosan films reduces the WVTR value. Clay silicate layers dispersed in the polymer matrix create a tortuous path for water molecules to enter, resulting in limited diffusion into the composites [215]. The addition of a plasticizer, glycerol, works depending on the concentration in which it is used. The addition of glycerol to 20% by weight reduces water sorption. This is related to the interaction between the hydroxyl groups of glycerol and the functional groups of chitosan. This leads to the formation of a network that prevents further interaction of water with the polar groups of chitosan. On the other hand, the addition of glycerol at a concentration of 30% by weight causes an increase in water absorption [215].

### 4.5. Optical Properties of Chitosan Films

In the food industry, UV radiation is used, among others, to sterilize food or to illuminate food store shelves in order to increase its attractiveness and encourage consumers to buy. Such actions expose packaged food, especially food sensitive to light, to photo-oxidation [216,217]. Literature data indicate that chitosan films show high light transmittance due to the lack of groups that could absorb UV-VIS radiation and that chitosan films are characterized by the transmission of 88–93% in the visible range (400–700 nm), while in the ultraviolet range (200–400 nm) the transmittance values range from 0–88% [218]. Literature data show that the inclusion of, inter alia, natural additives rich in phenolic compounds to the matrix of chitosan films increases their ability to absorb UV radiation, thus reducing their permeability [218,219]. Literature reports showed that the permeability of films, both in the visible and ultraviolet range, was reduced by adding additives to the chitosan film matrix, such as ellagic acid, lychee pericarp powder, or α-tocopherol [218,219,220,221]. Studies have shown that chitosan films with the addition of boswellic acid provide a very good UV barrier. UV light transmission of active films with the addition of boswellic acid decreased by 70% compared to foils without this additive, which may be due to the presence of the aromatic -OH group in boswellic acid, which increases the absorbance in the range of 200–400 nm [203]. Studies have shown that the inclusion of salicylic acid in the film matrix reduces its opacity, which is beneficial for food preservation [222]. Literature data show that additives such as gallic, gentisic, and protocatechuic acids also reduced the ultraviolet transmittance of chitosan films [223]. Furthermore, the addition of syringic acid acts as a good UV barrier. Studies have shown that the application of syringic acid reduced the ultraviolet transmittance of chitosan films in the 300–400 mm range. On the other hand, chitosan films with this addition showed much lower transparency [224].

Color measurement is also important for the functionality of the film due to its significant impact on its appearance. Studies show that chitosan films without additives are characterized by the highest transparency [170]. The transparency of the films is affected by the method of their preparation. Studies have shown that air-dried films are more transparent than heat-dried ones [214]. The use of additives such as cinnamaldehyde changes the color of the film. The changes in the color of films after the incorporation of additives into their matrix can be a visible indicator of chemical changes [189,225].

## 5. Biodegradation of Chitosan Films

One of the reasons it is so important to find a suitable alternative to plastics is their very long decomposition time and their harmfulness to the environment. Literature data showed that chitosan films become brittle and change color after a week, indicating the start of their degradation [226]. The degradation of chitosan films obtained with the use of various solvents and the influence of cross-linking on their degradation was described by Pavoni et al. [226]. The degradation process for all samples started within 90 days, the period in which plastics must disintegrate to be considered biodegradable according to ISO 20200: 2015. Moreover, the analysis of the ecotoxicity of organic compost samples used for the degradation of chitosan films showed that plant growth was not inhibited, which suggests the possibility of composting the film [226,227]. Research on chitosan film degradation was also carried out using three types of soil–industrial compost, commercial horticultural soil, and soil derived from a vineyard, with different water, organic, and inorganic matter content [228]. Chitosan films and films with the addition of *Quercus L.* polyphenol extract were subject to degradation. As a result of chemical destruction and the influence of animate or inanimate factors, chitosan films break down mechanically. Then mineralization by microorganisms takes place. Some materials may also degrade into smaller fragments under the influence of UV or thermal radiation. The degradation of chitosan films was faster than films with the addition of *Quercus L.* polyphenol extract, regardless of the conditions and environment of biodegradation, which may be related to the increased antioxidant and microbiological activity of chitosan films by introducing an additive into their matrix [228].

## 6. Conclusions and Further Perspectives

Currently, packaging materials are mainly made of plastics, which poses a serious threat to the environment. The observed environmental degradation, increased awareness, and consumer expectations make many industries, especially the packaging industry, face the necessity to introduce many changes. One of them is the introduction of new and biodegradable materials.

Technological progress and a better understanding of natural biopolymers and the possibilities of their use in the production of films mean that packaging materials made of natural raw materials, such as chitosan, have the potential as a biodegradable material that can replace plastics, e.g., in the packaging industry. Chitosan is a valuable raw material due to many valuable properties, such as biodegradability, non-toxicity, or antimicrobial properties, which make its use in the packaging industry of wide interest among researchers. An undoubted advantage of the material produced based on chitosan is the possibility of combining it with natural additives possessing antibacterial and antioxidant properties, which can improve the durability and safety of packaged food. At the same time, after their incorporation into the film matrix, they remain biodegradable and environmentally friendly. In the context of the packaging industry, chitosan films can be particularly useful for packaging food with a short and medium shelf life, such as meat, fish, or fruit, where, by including antibacterial additives, it is possible to prevent their faster spoilage. An important aspect of chitosan films, when used in the packaging industry, is their biodegradability. This can be a very beneficial aspect in the case of the disposal of packaging containing food residues or expired products. In the case of plastics that contain food residues, their recycling becomes difficult or impossible. Biodegradable films can be an interesting solution to this problem.

Despite the many advantages of natural packaging materials, they have inferior mechanical and barrier properties compared to traditional plastic packaging. Many natural additives improve these properties, while sometimes, the improvement of some properties is associated with the deterioration of others. More research is needed in this area to determine the appropriate concentrations of additives in the context of appropriate film applications. There is a need for more extensive research into the properties and use of additives to meet optical, mechanical, and barrier requirements. Moreover, an important aspect of packaging materials is knowledge about their biodegradability and disposal. Unfortunately, in review and research works, this aspect is often overlooked; it is related to the need to conduct extensive research or to consider that these materials are biodegradable if the components used for their production are so. Therefore, more research is needed in this area. In addition, it should be considered whether the new biodegradable packaging is economically viable and whether it is possible to produce them on a large scale. Above all, the problem of the process of obtaining chitosan itself should be considered. It is still a process that requires a lot of energy and generates a large amount of wastewater that must be properly treated and neutralized.

The review article indicated that chitosan films with natural additives were characterized by improved biological, mechanical, and physical properties, which suggests their use in the food packaging industry as a substitute for commonly used plastics. Moreover, the research showed that applying this natural biopolymer in food packaging extends the shelf-life of various foods and agricultural products, while simultaneously reducing the use of synthetic plastics, which in turn will positively impact the natural environment. However, further research on chitosan and its combinations with various materials is still needed to extend the application of chitosan in food packaging and bring its application to industrial levels.

## Figures and Tables

**Table 1:** The list of the most common used essential oils and their influence on the properties of the obtained chitosan-based films.

Essential Oils	Property	Results	Reference
Oregano *(Origanum vulgare)*	Antimicrobial activity	Chitosan-gelatin films incorporated with oregano essential oil showed inhibitory effects against *Escherichia coli*, *Staphylococcus aureus*, *Bacillus subtilis*, *Salmonella enteritidis,* and *Shiga bacillus.*	[88]
		The addition of oregano essential oil into the chitosan matrix improved antimicrobial activity against *S. aureus*, *Bacillus cereus*, *Salmonella typhimurium*, *E. coli*, *Candida albicans,* and *Aspergillus niger.*	[89]
	Mechanical strength	The addition of oregano oil had a negative effect on the mechanical strength of the obtained chitosan films.	[86]
		The addition of oregano essential oil significantly influenced the elongation at the break of films.	[90]
Cinnamon *(Cinnamonum Scheffer)*	Antimicrobial activity	Incorporation of cinnamon essential oil into chitosan-based films at higher than 0.4% exhibited a clear inhibitory zone by the absence of bacterial (*Lactobacillus plantarum*, *Lactobacillus sakei*, *Listeria monocytogenes*, *Pseudomonas fluorescens,* and *E. coli)* growth around the film cuts.	[91]
		The addition of cinnamon essential oil improved the antimicrobial activity of chitosan films against *E. coli*, *S. aureus*, *Penicillium digitatum,* and *Aspergillus oryzae*.	[92]
	Mechanical strength	The inclusion of cinnamon essential oil in the chitosan films significantly increased the tensile strength values and decreased elongation at break values.	[91]
Clove *(Syzygium aromaticum)*	Antimicrobial activity	Chitosan films with the addition of clove essential oil were effective against *L. monocytogenes* and *S. aureus*.	[92]
		The addition of clove essential oil improved the antimicrobial activity of chitosan films against *E. coli*, *S. aureus*, *P. digitatum,* and *A. oryzae*.	[93]
	Mechanical strength	The addition of clove essential oil caused the loss of mechanical strength of chitosan films.	[93]
Rosemary *(Rosmarinus officinalis)*	Antimicrobial activity	The addition of rosemary oil to chitosan film works best against gram-positive bacteria, especially against bacteria strains *L. monocytogenes* and *Streptococcus agalactiae.*	[94]
	Mechanical strength	The inclusion of rosemary essential oil in the chitosan films increased the tensile strength values and elongation at break.	[95]
Sage *(Salvia officinalis)*	Mechanical strength	The inclusion of sage essential oil in the chitosan films increased the tensile strength values.	[95]

**Table 2:** The list of the most common used plant extracts and their influence on the properties of the obtained films.

Plant Extracts	Property	Results	Reference
Rosemary *(Rosmarinus officinalis)*	Antimicrobial activity	The addition of rosemary extract into the film matrix increased antibacterial activity against *E. coli* and *S. aureus.*	[101]
	Antioxidant activity	The addition of rosemary extract into the film matrix increased the antioxidant activity.	[101]
	Mechanical strength	The mechanical strength of chitosan-based film depends on the amount of rosemary extract which was used. The mechanical strength of the film increases with the addition of rosemary extract, but at a certain point; as the extract content in the film increases, its strength begins to decline.	[101]
Turmeric *(Curcuma longa L.)*	Antimicrobial activity	Antimicrobial activity against *S. aureus* and *S. typhimurium* was improved by the addition of turmeric extract into the chitosan matrix.	[102]
		The addition of turmeric extract reduced the growth of *Botrytis cinerea* fungus.	[103]
	Mechanical strength	The incorporation of turmeric extract into chitosan films significantly increased the tensile strength values.	[102]
Thyme *(Thymus vulgaris)*	Antimicrobial activity	The addition of thyme extract into the film matrix increased antibacterial activity against *E. coli* and *S. aureus.*	[104]
	Antioxidant activity	The addition of thyme extract increased the antioxidant activity of the films.	[104]
	Mechanical strength	The incorporation of thyme extract into chitosan films decreased elongation at the break of the films.	[104]
Pomegranate rind *(Punica granatum)*	Antioxidant activity	The addition of pomegranate rind extract increased the antioxidant activity of the films.	[104]
	Mechanical strength	The incorporation of pomegranate rind extract into chitosan films increased the tensile strength values.	[105]
Green tea *(Camellia sinensis)*	Antimicrobial activity	The addition of green tea extract to the chitosan film matrix had a positive effect on its antibacterial properties against *E. coli* and *Listeria innocua.*	[106]
	Antioxidant activity	The addition of green tea extract increased the antioxidant activity of the films.	[107]
		The DPPH radical scavenging capacity of chitosan-based films was enhanced when green tea extract was added to the film matrix.	[108]
	Mechanical strength	The tensile strength and elongation at break were significantly decreased by the addition of green tea extract into chitosan films.	[107]

## Data Availability

No experimental data are reported in the review manuscript.
